# The optimal glycemic target in critically ill patients: an updated network meta-analysis

**DOI:** 10.1186/s40560-024-00728-0

**Published:** 2024-04-14

**Authors:** Aiko Tanaka, Tomoaki Yatabe, Tomohiro Suhara, Moritoki Egi

**Affiliations:** 1 Department of Intensive Care, University of Fukui Hospital, 23-3 Matsuoka Shimoaizuki, Eiheiji-Cho, Yoshida, Fukui 910-1193 Japan; 2 Department of Anesthesiology and Intensive Care Medicine, Osaka University Graduate School of Medicine, 2-15 Yamadaoka, Suita, Osaka 565-0871 Japan; 3 Emergency Department, Nishichita General Hospital, 3-1-1, Nakanoike, Tokai, Aichi 477-8522 Japan; 4 Department of Anesthesiology, Keio University School of Medicine, 35 Shinanomachi, Shinjuku-Ku, Tokyo, 160-8582 Japan; 5 Department of Anesthesia, Kyoto University Hospital, 54 Shogoin-Kawahara-Cho, Sakyo-Ku, Kyoto, 606-8507 Japan

**Keywords:** Glycemic control, Blood glucose, Optimal target, Critical care, Network meta-analysis

## Abstract

Acute glycemic control significantly affects the clinical outcomes of critically ill patients. This updated network meta-analysis examines the benefits and harms of four target blood glucose levels (< 110, 110–144, 144–180, and > 180 mg/dL). Analyzing data of 27,541 patients from 37 trials, the surface under the cumulative ranking curve for mortality and hypoglycemia was highest at a target blood glucose level of 144–180 mg/dL, while for infection and acute kidney injury at 110–144 mg/dL. Further evidence is needed to determine whether 110–144 or 144–180 mg/dL is superior as an optimal glucose target, considering prioritized outcomes.

**Supplementary Information:**

The online version contains supplementary material available at 10.1186/s40560-024-00728-0.

## Background

Though multiple studies have compared levels of glycemic control in critically ill patients, the optimal target blood glucose levels remain uncertain. The benefits of intensive insulin therapy (IIT) on patient outcomes were reported in 2001 [1], though conflicting results were reported in a recent large randomized controlled trial (RCT) [2]. This updated network meta-analysis was conducted to compare the benefits and harms of acute glycemic control and target blood glucose levels, including the results of a novel large-scale study.

## Methods

As this is an updated network meta-analysis of a previous study [3], a comprehensive search for English or Japanese RCTs in the PubMed, Cochrane Library databases, and ICHUSHI (April 1, 2019 to October 22, 2023) was conducted to identify additional studies regarding glycemic control in critically ill patients (Additional file 1). This study was registered in the University Hospital Medical Information Network Clinical Trials Registry (ID: 000049483). The titles and abstracts of the identified studies were independently screened by three investigators. Studies reporting primary data regarding adult patients treated in the intensive care unit were included if they compared different blood glucose target levels and reported outcome measures. The primary outcome of this study was hospital mortality. Secondary outcomes were 28- or 30-day mortality, long-term mortality, risk of infection resulting in sepsis, hypoglycemia (blood glucose levels < 40 mg/dL), and acute kidney injury (AKI; as defined by each author). The patients were divided into four groups based on the upper limit of the target blood glucose level: < 110, 110–144, 144–180, or > 180 mg/dL [3, 4]. A network meta-analysis was conducted to identify the optimal target blood glucose levels within a Bayesian framework using JAGS (version 4.3.0; SourceForge, San Diego, CA) and R (version 4.0.4; R Foundation for Statistical Computing, Vienna, Austria) software and the rjags and gemtc packages.

## Results

The data of 27,541 patients from 37 studies were included in this network meta-analysis (Additional files 2 and 3). The risk of bias for eligible studies is shown in Additional file 4 and network plots correlating target blood glucose levels with study outcomes are described in Additional file 5. The target blood glucose level of 144–180 mg/dL had the highest surface under the cumulative ranking curve (SUCRA) for hospital mortality (68.6), long-term mortality (70.1), 28- or 30-day mortality (78.3), and hypoglycemia (82.1) (Table 1, Additional file 6), while a target blood glucose level of 110–144 mg/dL had the highest SUCRA for infection (87.4) and AKI (89.8) (Table 1, Additional file 6). The absolute differences of infection and AKI between a target blood glucose level of 144–180 and 110–144 mg/dL were 29 more per 1000 (95% credible interval [CrI]: 58 fewer–131 more) and 118 more per 1000 (95% CrI: 82 fewer–473 more), respectively. On the other hand, the hypoglycemia of a target blood glucose level of 144–180 mg/dL was 65 fewer per 1000 (95% CrI: 81 fewer–9 more) than that of a target blood glucose level of 110–144 mg/dL.

**Table 1:** Absolute difference and ranking according to blood glucose level

	Hospital mortality		28- or 30-day mortality		Long-term mortality		Infection		Hypoglycemia	
	Absolute difference (95% CrI)	Rank (SUCRA)	Absolute difference (95% CrI)	Rank (SUCRA)	Absolute difference (95% CrI)	Rank (SUCRA)	Absolute difference (95% CrI)	Rank (SUCRA)	Absolute difference (95% CrI)	Rank (SUCRA)
vs < 110 mg/dL										
< 110mg/dL	No estimable	3 (47.4)	No estimable	2 (49.7)	No estimable	4 (34.2)	No estimable	3 (42.4)	No estimable	4 (14.3)
110–144 mg/dL	15 fewer per 1000 (73 fewer to 70 more)	2 (65.9)	40 more per 1000 (88 fewer to 198 more)	4 (28.9)	14 fewer per 1000 (123 fewer to 159 more)	2 (55.3)	35 fewer per 1000 (82 fewer to 27 more)	1 (87.4)	14 fewer per 1000 (90 fewer to 273 more)	3 (21.6)
144–180 mg/dL	16 fewer per 1000 (79 fewer to 38 more)	1 (68.6)	11 fewer per 1000 (40 fewer to 19 more)	1 (78.3)	15 fewer per 1000 (48 fewer to 18 more)	1 (70.1)	13 fewer per 1000 (44 fewer to 20 more)	2 (69.0)	69 fewer per 1000 (79 fewer to 49 fewer)	1 (82.1)
> 180 mg/dL	10 more per 1000 (13 fewer to 42 more)	4 (18.0)	4 more per 1000 (34 fewer to 50 more)	3 (43.1)	1 fewer per 1000 (19 fewer to 21 more)	3 (40.4)	31 more per 1000 (4 more to 68 more)	4 (1.2)	48 fewer per 1000 (54 fewer to 39 fewer)	2 (81.9)
vs 110–144 mg/dL										
144–180 mg/dL	4 more per 1000 (96 fewer to 120 more)		43 fewer per 1000 (135 fewer to 83 more)		1 more per 1000 (59 fewer to 122 more)		29 more per 1000 (58 fewer to 131 more)		65 fewer per 1000 (81 fewer to 9 more)	
> 180 mg/dL	38 more per 1000 (53 fewer to 151 more)		28 fewer per 1000 (122 fewer to 99 more)		8 more per 1000 (56 fewer to 130 more)		109 more per 1000 (19 more to 218 more)		28 fewer per 1000 (35 fewer to 10 more)	
vs 144–180 mg/dL										
> 180 mg/dL	2 more per 1000 (1 fewer to 8 more)		1 more per 1000 (1 fewer to 4 more)		13 more per 1000 (23 fewer to 57 more)		26 more per 1000 (4 more to 61 more)		0 fewer per 1000 (6 fewer to 16 more)	

	Acute kidney injury	
	Absolute difference (95% CrI)	Rank (SUCRA)
vs < 110 mg/dL		
< 110 mg/dL	No estimable	2 (46.7)
110–144 mg/dL	34 fewer per 1000 (64 fewer to 27 more)	1 (89.8)
144–180 mg/dL	10 more per 1000 (66 fewer to 184 more)	3 (36.6)
> 180 mg/dL	8 more per 1000 (23 fewer to 52 more)	4 (27.0)
vs 110–144 mg/dL		
144–180 mg/dL	118 more per 1000 (82 fewer to 473 more)	
> 180 mg/dL	120 more per 1000 (17 fewer to 324 more)	
vs 144–180 mg/dL		
> 180 mg/dL	0 more per 1000 (67 fewer to 164 more)	

*CrI* credible interval, *SUCRA* surface under the cumulative ranking

## Discussion

Mortality rates were not significantly different between the four target blood glucose levels, while the risk of infection increased when the target blood glucose level was > 180 mg/dL. The risk of hypoglycemia did not differ significantly when a target blood glucose level of 144–180 or > 180 mg/dL was used. The risk of hypoglycemia was fivefold higher with target blood glucose levels of < 110 or 110–144 mg/dL compared to a target blood glucose level of 144–180 mg/dL. Therefore, a target blood glucose level of 144–180 mg/dL may be the better harm–benefit balance, especially in terms of avoiding hypoglycemia.

The largest study included in this meta-analysis (*n* = 9230 patients) [2] compared IIT (target range: 80–110 mg/dL) with liberal glycemic control (180–215 mg/dL) without parenteral nutrition within 7 days, reflecting the current guidelines for clinical nutrition [5]. Despite the use of a high-performance computer algorithm to reduce the incidence of hypoglycemia, IIT did not demonstrate advantages in terms of mortality compared to the liberal glycemic control group. Only four RCT used a computer-guided glucose control device (we marked asterisk in Additional file 3). Therefore, we could not conduct subgroup analysis. However, the incidence of hypoglycemia in the 80–110 mg/dL group in this RCT was 1%, which was lower than that in the two previous RCTs (11%) [1, 6] conducted at the same center and in the NICE-SUGAR study (7%) [7]. This result indicated that the balance of benefits and harms between groups might change when a computer-guided glucose control device is used.

In addition, this RCT showed significantly better results in the incidence of AKI and liver dysfunction in the 80–110 mg/dL group. Although we could not perform a network meta-analysis because liver dysfunction was reported in five RCTs, our meta-analysis also showed that the risk of AKI using a target blood glucose level of 110–144 mg/dL may be lower than that of a target blood glucose level of 144–180 mg/dL. These data provide a new hypothesis that a target blood glucose level of 110–144 mg/dL without hypoglycemia is optimal in terms of less organ damage.

Observational studies have reported that critically ill patients with diabetes, which affects glucose metabolism, have different thresholds for the harmful effects of hyperglycemia than those without diabetes [8, 9]. In our meta-analysis, there were two RCTs conducted in only diabetic patients, and five RCTs reported sub-analyses focused on the diabetic patients. Of these, a pairwise meta-analysis about hospital or 90-day mortality was able to be performed using three RCTs (Additional file 7). This result revealed that at least glycemic control aiming 80–110 mg/dL might not be suitable to diabetes patients. Therefore, data regarding glycemic control in patients with diabetes who are administered acute nutritional therapy are needed.

## Conclusions

Further evidence is needed to determine whether 110–144 or 144–180 mg/dL is superior as an optimal glucose target range. The harm–benefit balance will vary depending on which outcome is prioritized, particularly if the incidence of hypoglycemia is low using a computer-guided glucose control device.

### Supplementary Information

**Additional file 1.** Search strategy.

**Additional file 2.** Flow diagram of study selection.

**Additional file 3.** Characteristics of the studies included in the network meta-analysis.

**Additional file 4.** Risk of bias in the included randomized controlled trials.

**Additional file 5.** The network of all eligible comparisons for the meta-analysis.

**Additional file 6.** Estimates of effects, and certainty of the evidence according to blood glucose levels.

**Additional file 7.** Forest plot of pairwise comparison between intensive insulin therapy vs conventional care (more than 144mg/dL) for hospital or 90-days mortality in diabetes patients.

## Data Availability

The datasets of this meta-analysis are available from the corresponding author on reasonable request.

## Abbreviations

AKI: Acute kidney injury
CrI: Credible interval
IIT: Intensive insulin therapy
RCT: Randomized controlled trial
SUCRA: Surface under the cumulative ranking curve
