# Supplementary material for: The optimal glycemic target in critically ill patients: an updated network meta-analysis
Source: J Intensive Care. 2024 Apr 14;12:14. doi: 10.1186/s40560-024-00728-0 (PMC11017653; doi:10.1186/s40560-024-00728-0)
Supplement: Supplementary file 1 — Additional file 1. Search strategy. [file 40560_2024_728_MOESM1_ESM.docx]

**Additional file 1.** Search strategy.

| **PubMed** | |
| --- | --- |
| #1 | ((("intensive care units"[MeSH Terms] OR "intensive care unit" OR ICU) OR ("critical care"[MeSH Terms] OR "critical care") OR (sepsis[MeSH Terms] OR sepsis) OR (critical illness[MeSH Terms] OR "critical illness") OR ("Brain Ischemia/complications"[Mesh] OR "Brain Ischemia/diet therapy"[Mesh] OR "Brain Ischemia/drug therapy"[Mesh] OR "Brain Ischemia/therapy"[Mesh]))) |
| #2 | ((((("Blood Glucose/administration and dosage"[Mesh] OR "Blood Glucose/adverse effects"[Mesh] OR "Blood Glucose/drug effects"[Mesh] OR "Blood Glucose/therapeutic use"[Mesh])) OR ("Insulin/administration and dosage"[Mesh] OR "Insulin/adverse effects"[Mesh] OR "Insulin/therapeutic use"[Mesh] OR "Insulin/therapy"[Mesh]) OR (((("Insulin/administration and dosage"[Mesh] OR "Insulin/adverse effects"[Mesh] OR "Insulin/therapeutic use"[Mesh] OR "Insulin/therapy"[Mesh]))) OR insulin) OR ((("glycemic control") OR glycaemic control OR (glycemic AND control)) OR (glycaemic AND control))))) |
| #3 | ((randomized controlled trial[pt] OR controlled clinical trial[pt] OR randomized[tiab]) OR placebo[tiab] OR drug therapy[sh] OR randomly[tiab] OR trial[tiab] OR groups[tiab] NOT (animals[mh] NOT humans[mh])) |
| #4 | #1 AND #2 AND #3 |
| **CENTRAL** | |
| #1 | MeSH descriptor: [Intensive Care Units] explode all trees |
| #2 | (”intensive care unit”):ti,ab,kw |
| #3 | (ICU):ti,ab,kw |
| #4 | MeSH descriptor: [Critical Care] explode all trees |
| #5 | (”critical care”):ti,ab,kw |
| #6 | MeSH descriptor: [Sepsis] explode all trees |
| #7 | (sepsis):ti,ab,kw |
| #8 | MeSH descriptor: [Critical Illness] explode all trees |
| #9 | (”critical illness”):ti,ab,kw |
| #10 | MeSH descriptor: [Brain Ischemia] explode all trees |
| #11 | #1 OR #2 OR #3 OR #4 OR #5 OR #6 OR #7 OR #8 OR #9 OR #10 |
| #12 | MeSH descriptor: [Blood Glucose] explode all trees |
| #13 | MeSH descriptor: [Insulins] in all MeSH products |
| #14 | (insulin):ti,ab,kw |
| #15 | ("glycemic control"):ti,ab,kw |
| #16 | (glycemic):ti,ab,kw |
| #17 | (control):ti,ab,kw |
| #18 | #16 AND #17 |
| #19 | #12 OR #13 OR #14 OR #15 OR #16 OR #18 |
| **ICHUSHI** (Japanese) | |
| #1 | (((クリティカルケア/TH or 集中治療/TA)) and (PT=会議録除く)) |
| #2 | ((敗血症/TH or 敗血症/TA)) and (PT=会議録除く) |
| #3 | ((血糖/TH or 血糖/TA)) and (PT=会議録除く) |
| #4 | #1 or #2 |
| #5 | #3 and #4 |
| #6 | (ランダム化比較試験/TH) |
| #7 | (準ランダム化比較試験/TH) |
| #8 | (ランダム化/AL) |
| #9 | (無作為化/AL) |
| #10 | (比較試験/AL) |
| #11 | (臨床試験/AL) |
| #12 | (プラセボ/AL) |
| #13 | (対象/AL) |
| #14 | (コントロール/AL) |
| #15 | (臨床研究/AL) |
| #16 | #6 or #7 or #8 or #9 or #10 or #11 or #12 or #13 or #14 or #15 |
| #17 | #5 and #16 |
