# Supplementary figures and images for: The optimal glycemic target in critically ill patients: an updated network meta-analysis
Source: J Intensive Care. 2024 Apr 14;12:14. doi: 10.1186/s40560-024-00728-0 (PMC11017653; doi:10.1186/s40560-024-00728-0)

**Additional file 2.** Flow diagram of study selection.

**
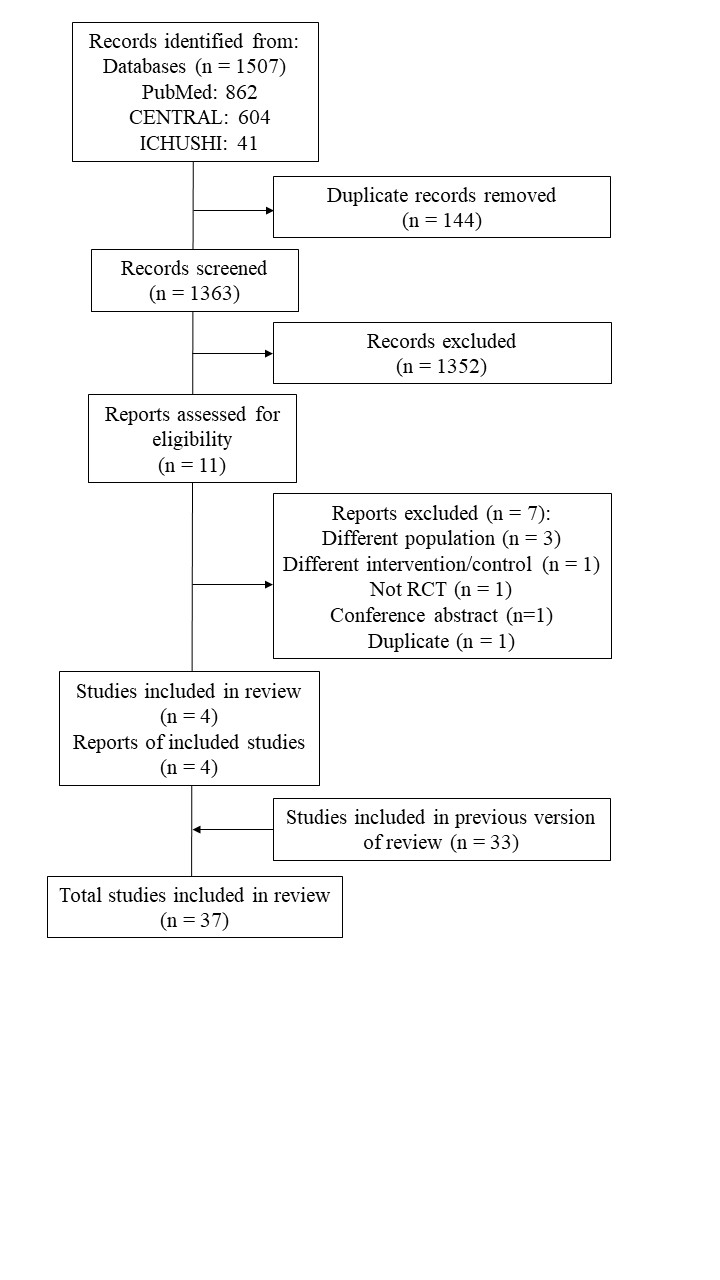
**

Supplement: Supplementary file 2 — Additional file 2. Flow diagram of study selection. [file 40560_2024_728_MOESM2_ESM.docx]

**
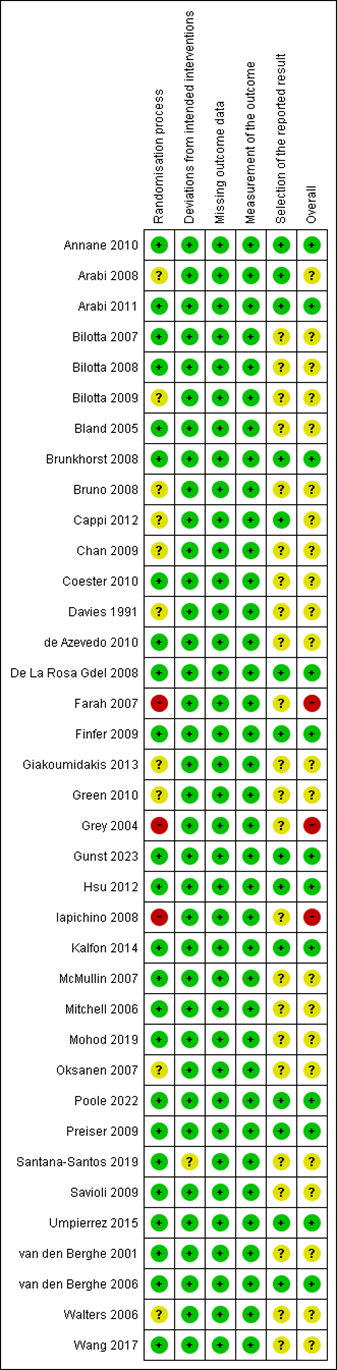
Additional file 4.** Risk of bias in the included randomized controlled trials.

Supplement: Supplementary file 4 — Additional file 4. Risk of bias in the included randomized controlled trials. [file 40560_2024_728_MOESM4_ESM.docx]
