# Supplementary material for: The optimal glycemic target in critically ill patients: an updated network meta-analysis
Source: J Intensive Care. 2024 Apr 14;12:14. doi: 10.1186/s40560-024-00728-0 (PMC11017653; doi:10.1186/s40560-024-00728-0)
Supplement: Supplementary file 3 — Additional file 3. Characteristics of the studies included in the network meta-analysis. [file 40560_2024_728_MOESM3_ESM.docx]

**Additional file 3.** Characteristics of the studies included in the network meta-analysis

| Author | Year | Sample size | Included patients | Glucose target (mg/dL) | Age | Severity | Outcome measured |
| --- | --- | --- | --- | --- | --- | --- | --- |
| Annane [1] | 2010 | 509 | Adults with septic shock who presented with multiple organ dysfunction | 80–110 | 63.7(95%CI 61.9–64.3) | SAPS II score:58.9 (56.9–60.0)  SOFA score: 10.4 (10–10.8) | Hospital mortality, 30 day mortality, long term mortality, infection. and hypoglycemia |
|  |  |  |  | 180–200 | 64.3 (62.4–66.1) | SAPS II score: 60.4 (58.2–62.6)  SOFA score:10.8 (10.3–112.2) |  |
| Arabi [2] | 2008 | 523 | Patients aged ≥18 y and had serum glucose level as measured by the laboratory of >110 mg/dL during the first 24 h of ICU admission  (Diabetes=40%) | 80–110 | 50.6±22.6 | APACHE II score: 22.5±7.9  SOFA score: 8.7±3.5 | Hospital mortality, infection, and hypoglycemia |
|  |  |  |  | 180–200 | 54.3±20.5 | APACHE II score: 23.1±8.4  SOFA score: 8.8±3.5 |  |
| Arabi [3] | 2011 | 240 | Patients aged ≥18 y with a blood glucose concentration of >110 mg/dL, receiving enteral feeding, and expected to stay for ≥48 h  (Diabetes=40%) | 80–110 | 53.0±21.3 | APACHE II score: 25.3±7.5  SOFA score: 9.9±3.0 | Hospital mortality, 28 day mortality, long term mortality, infection, and hypoglycemia |
|  |  |  |  | 180–200 | 49.3±22.0 | APACHE II score: 25.2±8.2  SOFA score: 10.6±3.5 |  |
| Bilotta [4] | 2007 | 78 | Patients with sub-arachnoid hemorrhage undergoing emergency cerebral aneurysm clipping with a prehospitalization Rankin disability score of 0 or 17 who were not endotracheally intubated and were admitted to neurosurgical ICU after surgery  (Diabetes=10%) | 80–120 | 53±16 |  | Long term mortality and infection |
|  |  |  |  | 80–220 | 52±15 |  |  |
| Bilotta [5] | 2008 | 97 | Patients who had TBI and GCS ≤8 requiring placement of an intracranial pressure monitor or evidence of traumatic mass lesion on computed tomographic scan Patients eligible for this study were expected to stay in the ICU for at least 3 days  (Diabetes=12%) | 80–120 | 52±16 | SAPS II score: 57.5 (42–70) | Hospital mortality, long term mortality, infection, and hypoglycemia |
|  |  |  |  | <220 | 53±15 | SAPS II score: 64 (50–76) |  |
| Bilotta [6] | 2009 | 483 | Patients aged ≥18 y and were consecutively admitted to the postoperative neurosurgical ICU after elective or emergency surgery  (Diabetes=10%) | 80–120 | 57.34±11.94 | SAPS II score: 41.73±18.85 | Long term mortality, infection, and hypoglycemia |
|  |  |  |  | 180–200 | 56.9±12.65 | SAPS II score: 42.36±21.33 |  |
| Bland [7] | 2005 | 10 | All patients admitted to the medical ICU who had been receiving mechanical ventilation for less than 24 hours  (Diabetes=40%) | 80–110 | 56.7±15.2 overall |  | 28 day mortality and hypoglycemia |
|  |  |  |  | 180–200 |  |  |  |
| Brunkhorst [8] | 2008 | 537 | Patients with severe sepsis or septic shock who were at least 18 years of age and treated in the ICU  (Diabetes=30%) | 80–110 | 64.0±14.3 | APACHE II score: 20.2±6.6 | 28 day mortality, long term mortality, hypoglycemia, and AKI |
|  |  |  |  | 180–200 | 65.2±13.2 | APACHE II score: 20.3±6.8 |  |
| Bruno [9] | 2008 | 46 | Ischemic stroke with onset of symptoms within 12 h before randomization, baseline blood glucose level 150 mg/dL, and baseline NIHSS score of 3 to 22, with at least 2 points on the motor portion  (Diabetes=91%) | 90–130 | 62±15 | NIHSS score: 9 (5–15) | Long term mortality, and hypoglycemia |
|  |  |  |  | <200 | 53±15 | NIHSS score: 10 (6–15) |  |
| Cappi [10] | 2012 | 63 | All patients aged >18 y, diagnosed at ICU admission with severe sepsis or septic shock  (Diabetes=24%) | 80–110 | 53±19 | APACHE II score: 19±7 | Hospital mortality, 28 day mortality, and hypoglycemia |
|  |  |  |  | 140–180 | 53±19 | APACHE II score: 19±7 |  |
| Chan [11] | 2009 | 109 | Adults aged >21 y and who were undergoing open-heart cardiac surgery with cardiopulmonary bypass  (Diabetes=29%) | 80–130 | 57±12 | EuroSCORE: 3.2±2.2 | 30 day mortality, infection, hypoglycemia, and AKI |
|  |  |  |  | 160– 200 | 58±12 | EuroSCORE: 3.5±2.1 |  |
| Coester [12] | 2010 | 79 | Patients with blunt traumatic brain injury aged ≥18 y, presenting with GCS ≤8 and admitted to the ICU  (Diabetes=1%) | 80–110 | 38.85±16.3 | APACHE II score: 15 (9.25–20.75) | Hospital mortality, long term mortality, infection, hypoglycemia, and AKI |
|  |  |  |  | <180 | 38.13±14.1 | APACHE II score: 16 (9–23) |  |
| Davies [13] | 1991 | 69 | Patients with suspected myocardial infarction known to have diabetes prior to admission to CCU  (Diabetes=100%) | 72–144 | 64±10 |  | Hospital mortality |
|  |  |  |  | <180 | 60±11 |  |  |
| de Azevedo [14] | 2010 | 337 | All adult, nonpregnant, patients admitted to ICUs, who had at least 2 blood glucose levels above 150 mg/dL from 3 measurements obtained in the first 12 h after admission  (Diabetes=31%) | 80–120 | 56.4±21.0 | APACHE II score: 67.8±24.1 | Infection and hypoglycemia |
|  |  |  |  | <180 (ideally <150 in stable patients) | 56.1±20.4 | APACHE II score: 66.9±29.0 |  |
| De La Rosa Gdel [15] | 2008 | 504 | Patients aged ≥15 y admitted to the ICU with an expected ICU stay of at least two days  (Diabetes=12%, sub-analyses focused on DM) | 80–110 | 45.9±20.2 | APACHE II score: 15.7±6.9  SOFA score: 7.3±3.2 | Hospital mortality, 28 day mortality, infection, hypoglycemia, and AKI |
|  |  |  |  | 180–200 | 47.4±19.3 | APACHE II score: 15.6 ± 7.6  SOFA score: 7.6 ± 3.5 |  |
| Farah [16] | 2007 | 89 | Patients admitted to the ICU  (Diabetes=60%) | 110–140 | 71.86±14.07 | APACHE II score: 22.3±4.2 | Hospital mortality, 28 day mortality, infection, and AKI |
|  |  |  |  | 140–200 | 74.21±12.74 | APACHE II score: 21.9 ± 4.4 |  |
| Finfer [17] | 2009 | 6,030 | Patients expected to require treatment in the ICU on 3 or more consecutive days  (Diabetes=20%, sub-analyses focused on DM) | 81–108 | 60.4±17.2 | APACHE II score: 21.1±7.91 | 28 day mortality, long term mortality, infection, and hypoglycemia |
|  |  |  |  | <180 | 59.9±17.1 | APACHE II score: 21.1±8.3 |  |
| Giakoumidakis [18] | 2013 | 212 | (1) open heart surgery, (2) surgery requiring CPB, (3) patient aged ≥18 y (4) the patient’s informed consent for participation in our study  (Diabetes=28%) | 120–160 | 64.9±11.5 |  | Hospital mortality, 30 day mortality, infection, and hypoglycemia |
|  |  |  |  | 161–200 | 66.9±11.1 |  |  |
| Green [19]* | 2010 | 81 | Mechanically ventilated neurologic patients expected to have an ICU length of stay of at least 3 days | 80–110 | 50±22 |  | Long term mortality, infection, and hypoglycemia |
|  |  |  |  | ≤150 | 51±18 |  |  |
| Grey [20] | 2004 | 61 | Adult patients admitted to a 12-bed general surgical ICU who required treatment for hyperglycemia  (Diabetes=39%) | 80–120 | 56±22 | APACHE II score: 15.6±6.1 | Hospital mortality, and infection |
|  |  |  |  | 180–220 | 55±22 | APACHE II score: 15.1±6.5 |  |
| Hsu [21] | 2012 | 112 | Aged ≥18 y, admission to the medical ICU with blood glucose >180mg/dL  (Diabetes=40%) | 120–140 | 68.1±16.3 | APACHE II score (median (range)): 20 (17–25) | Hospital mortality, infection, hypoglycemia, and AKI |
|  |  |  |  | 180–200 | 70.4±12.1 | APACHE II score (median (range)): 21 (16.5–26.5) |  |
| Iapichino [22] | 2008 | 90 | Those who met the criteria for severe sepsis  (Diabetes=17%) | 80–110 | 60.3±14.2 | SAPS II score: 41.3±14.3  SOFA score: 9.3±2.8 | Long term mortality, and hypoglycemia |
|  |  |  |  | 180–200 | 64.3±14.3 | SAPS II score: 42.2±12.2  SOFA score: 8.7±3.3 |  |
| Kalfon [23]* | 2014 | 2,864 | Adult patients assumed to require ≥ 3 days in medical, surgical or mixed medical–surgical ICUs  (Diabetes=20%) | 80–110 | 61±16 | SAPS II score: 51±19 | Hospital mortality, 30 day mortality, long term mortality, infection, and hypoglycemia |
|  |  |  |  | ≤180 | 62±16 | SAPS II score: 51±19 |  |
| McMullin [24] | 2007 | 20 | Adult ICU patients if they were at least 18 years of age, expected to be in ICU for at least 72 h, and had a glucose value of at least 10 mmol/L within 48 h of ICU admission  (Diabetes=70%) | 90–126 | 69.4±15.0 | APACHE II score: 31.5±10.0 | Hospital mortality, infection, hypoglycemia, and AKI |
|  |  |  |  | 144–180 | 68.1±8.1 | APACHE II score: 32.0±11.0 |  |
| Mitchell [25] | 2006 | 70 | Patients aged ≥18 y who had been admitted to the ICU, and who were predicted at the time of ICU admission to stay more than 8 h in the ICU  (Diabetes=14%) | 72–108 | 66.28 (59.5–74.9) | APACHE II score: 19 (16–23) | Hospital mortality and hypoglycemia |
|  |  |  |  | 108–144 | 64.6 (57.7–73.1) | APACHE II score: 22 (20–29) |  |
| Oksanen [26] | 2007 | 90 | All consecutive patients admitted to the participating ICUs after successful resuscitation from OHCA were screened. Patients with VF of presumably cardiac origin, witnessed arrest, aged ≥18 y, basic life support delay of less than 15 min, ROSC in less than 35 min, and unresponsive at hospital admission were eligible for enrolment.  (Diabetes=11%) |  | 66 (52–72) | APACHE II score: 26 (18–29)  SAPS II score: 49 (39–61) | 30 day mortality and hypoglycemia |
|  |  |  |  |  | 62 (56–70) | APACHE II score: 24 (19–33)  SAPS II score: 47 (32–63) |  |
| Preiser [27] | 2009 | 1,101 | Adult patients (aged ≥18 y) admitted to the participating ICUs  (Diabetes=19%) | 80–110 | 64.8 (50.8–74.0) | APACHE II score: 15 (11–21)  SOFA score: 6.7±3.3 | Hospital mortality, 28 day mortality, and hypoglycemia |
|  |  |  |  | 140–180 | 64.5 (51.1–74.1) | APACHE II score: 15 (11–21)  SOFA score: 6.9±3.1 |  |
| Savioli [28] | 2009 | 90 | Sepsis patients based on 2001 criteria  (Diabetes=13%) | 80–110 | 58±15 | SAPS II score: 40.8±15.1  SOFA score: 10.7±3.5 | Long term mortality |
|  |  |  |  | 180–200 | 64±14 | SAPS II score: 44.1±14.5  SOFA score: 10.3±3.7 |  |
| Umpierrez [29]* | 2015 | 302 | Patients with and without diabetes undergoing primary, elective, and emergency CABG who experienced perioperative hyperglycemia, defined as a blood glucose>140 mg/dL.  Patients aged between 18 and 80 years undergoing primary or a combination of CABG and other cardiac operations such as valve repair or aortic surgery  (Diabetes=50%) | 100–140 | 64±9 | APACHE II score: 21.7±3.6 | Hypoglycemia |
|  |  |  |  | 141–180 | 64±10 | APACHE II score: 22.4±3.3 |  |
| van den Berghe [30] | 2001 | 1,548 | All adults receiving mechanical ventilation who were admitted to the ICU  (Diabetes=13%, sub-analyses focused on DM) | 80–110 | 63.4±13.6 | APACHE II score: 9 (7–13) for both groups | Hospital mortality, infection, and hypoglycemia |
|  |  |  |  | 180–200 | 62.2±13.9 |  |  |
| van den Berghe [31] | 2006 | 1,200 | Adult patients admitted to the medical ICU who were assumed to require at least a third day of intensive care  (Diabetes=17%, sub-analyses focused on DM) | 80–110 | 63±16 | APACHE II score: 23±10 | Hospital mortality, 28 day mortality, long term mortality, hypoglycemia, and AKI |
|  |  |  |  | 180–200 | 64±16 | APACHE II score: 23±9 |  |
| Walters [32] | 2006 | 25 | Patients admitted to Acute Stroke Unit within 24 h of onset of CT-confirmed ischemic stroke, venous blood glucose greater than 8 mmol/l and GCS >8  (Diabetes=52%) | 90–144 | 73.3±12.5 |  | Hospital mortality and hypoglycemia |
|  |  |  |  | ≤270 | 76.7±9.5 |  |  |
| Wang [33] | 2017 | 88 | Patients with TBI aged ≥18 y, a GCS score <8, and admission to the postoperative neurosurgical ICU  (Diabetes=19%) | 80–110 | 46.7±10.4 | APACHE II score: 28.6±11.7 | Hospital mortality, long term mortality, and infection |
|  |  |  |  | 180–200 | 45.1±10.7 | APACHE II score: 28.3±10.9 |  |
| Mohod [34] | 2019 | 40 | Adult diabetic and non‑diabetic patients posted for on‑pump CABG  (Diabetes=40%) | 80–110 | 54.1±9.9 |  | Mortality, infection, hypoglycemia, and AKI |
|  |  |  |  | ≤200 | 56.1±8.0 |  |  |
| Poole [35] | 2022 | 419 | Adult patients with type 2 diabetes expected to be in the ICU on at least three consecutive days  (Diabetes=100%) | 108–180 | 66 (58–73) | APACHE II score: 20 (16–26)  SOFA score: 7 (6–10) | Long term mortality, infection, and hypoglycemia |
|  |  |  |  | 180–252 | 67 (95%CI 58–75) | APACHE II score: 20 (16–26)  SOFA score: 8 (6–10) |  |
| Santana-Santos [36] | 2019 | 95 | Patients undergoing cardiac surgery, who presented glycemia ≥200 mg/dL in the first 6 h of ICU admission  (Diabetes=42%) | 90–110 | 60±12.5 | SOFA score: 11.7±1.1 | Hospital mortality, hypoglycemia, and AKI |
|  |  |  |  | 140–180 | 59.7±13.2 | SOFA score: 11.8±1.3 |  |
| Gunst [37]* | 2023 | 9,230 | Adult patients (aged ≥18 y) admitted to one of the participating ICUs  (Diabetes=20%, sub-analyses focused on DM) | 80–110 | 67 (57–75) | APACHE II score: 21 (15–30) | Hospital mortality, long term mortality, hypoglycemia, and AKI |
|  |  |  |  | 180–215 | 67 (56–75) | APACHE II score: 21 (15–30) |  |
| Data are presented the mean ± standard deviation or the median and interquartile range, unless otherwise noted.  APACHE, acute physiology and chronic health evaluation; CABG, coronary artery bypass grafting; CCU, Coronary Care Unit; CI, confidence interval; CPB, cardio-pulmonary bypass; CT, computed tomography; GCS, Glasgow coma scale; EuroSCORE, European system for cardiac operative risk evaluation; ICU, intensive care unit; NIHSS, National Institutes of Health Stroke Scale; OHCA, out-of-hospital cardiac arrest; ROSC, return of spontaneous circulation; SAPS, simplified acute physiology score; SOFA, sequential organ failure assessment; TBI, traumatic brain injury; VF, ventricular fibrillation; AKI, acute kidney injury  *: using a computer-guided glucose control device  sub-analyses focused on DM: RCT reported sub-analyses focused on the diabetic patients. | | | | | | | |

**References**

1. The COIITSS Study Investigators, Annane D, Cariou A, Maxime V, Azoulay E, D'Honneur G, et al. Corticosteroid treatment and intensive insulin therapy for septic shock in adults: a randomized controlled trial. JAMA. 2010;303:341-8.

2. Arabi YM, Dabbagh OC, Tamim HM, Al-Shimemeri AA, Memish ZA, Haddad SH, et al. Intensive versus conventional insulin therapy: a randomized controlled trial in medical and surgical critically ill patients. Crit Care Med. 2008;36:3190-7.

3. Arabi YM, Tamim HM, Dhar GS, Al-Dawood A, Al-Sultan M, Sakkijha MH, et al. Permissive underfeeding and intensive insulin therapy in critically ill patients: a randomized controlled trial. Am J Clin Nutr. 2011;93:569-77.

4. Bilotta F, Spinelli A, Giovannini F, Doronzio A, Delfini R, Rosa G. The effect of intensive insulin therapy on infection rate, vasospasm, neurologic outcome, and mortality in neurointensive care unit after intracranial aneurysm clipping in patients with acute subarachnoid hemorrhage: a randomized prospective pilot trial. J Neurosurg Anesthesiol. 2007;19:156-60.

5. Bilotta F, Caramia R, Cernak I, Paoloni FP, Doronzio A, Cuzzone V, et al. Intensive insulin therapy after severe traumatic brain injury: a randomized clinical trial. Neurocrit Care. 2008;9:159-66.

6. Bilotta F, Caramia R, Paoloni FP, Delfini R, Rosa G. Safety and efficacy of intensive insulin therapy in critical neurosurgical patients. Anesthesiology. 2009;110:611-9.

7. Bland DK, Fankhanel Y, Langford E, Lee M, Lee SW, Maloney C, et al. Intensive versus modified conventional control of blood glucose level in medical intensive care patients: a pilot study. Am J Crit Care. 2005;14:370-6.

8. Brunkhorst FM, Engel C, Bloos F, Meier-Hellmann A, Ragaller M, Weiler N, et al. Intensive insulin therapy and pentastarch resuscitation in severe sepsis. N Engl J Med. 2008;358:125-39.

9. Bruno A, Kent TA, Coull BM, Shankar RR, Saha C, Becker KJ, et al. Treatment of hyperglycemia in ischemic stroke (THIS): a randomized pilot trial. Stroke. 2008;39:384-9.

10. Cappi SB, Noritomi DT, Velasco IT, Curi R, Loureiro TC, Soriano FG. Dyslipidemia: a prospective controlled randomized trial of intensive glycemic control in sepsis. Intensive Care Med. 2012;38:634-41.

11. Chan RP, Galas FR, Hajjar LA, Bello CN, Piccioni MA, Auler JO, Jr. Intensive perioperative glucose control does not improve outcomes of patients submitted to open-heart surgery: a randomized controlled trial. Clinics (Sao Paulo). 2009;64:51-60.

12. Coester A, Neumann CR, Schmidt MI. Intensive insulin therapy in severe traumatic brain injury: a randomized trial. J Trauma. 2010;68:904-11.

13. Davies RR, Newton RW, McNeill GP, Fisher BM, Kesson CM, Pearson D. Metabolic control in diabetic subjects following myocardial infarction: difficulties in improving blood glucose levels by intravenous insulin infusion. Scott Med J. 1991;36:74-6.

14. de Azevedo JR, de Araujo LO, da Silva WS, de Azevedo RP. A carbohydrate-restrictive strategy is safer and as efficient as intensive insulin therapy in critically ill patients. J Crit Care. 2010;25:84-9.

15. De La Rosa Gdel C, Donado JH, Restrepo AH, Quintero AM, Gonzalez LG, Saldarriaga NE, et al. Strict glycaemic control in patients hospitalised in a mixed medical and surgical intensive care unit: a randomised clinical trial. Crit Care. 2008;12:R120.

16. Farah R, Samokhvalov A, Zviebel F, Makhoul N. Insulin therapy of hyperglycemia in intensive care. Isr Med Assoc J. 2007;9:140-2.

17. NICE-SUGAR Study Investigators, Finfer S, Chittock DR, Su SY, Blair D, Foster D, et al. Intensive versus conventional glucose control in critically ill patients. N Engl J Med. 2009;360:1283-97.

18. Giakoumidakis K, Eltheni R, Patelarou E, Theologou S, Patris V, Michopanou N, et al. Effects of intensive glycemic control on outcomes of cardiac surgery. Heart Lung. 2013;42:146-51.

19. Green DM, O'Phelan KH, Bassin SL, Chang CW, Stern TS, Asai SM. Intensive versus conventional insulin therapy in critically ill neurologic patients. Neurocrit Care. 2010;13:299-306.

20. Grey NJ, Perdrizet GA. Reduction of nosocomial infections in the surgical intensive-care unit by strict glycemic control. Endocr Pract. 2004;10 Suppl 2:46-52.

21. Hsu CW, Sun SF, Lin SL, Huang HH, Wong KF. Moderate glucose control results in less negative nitrogen balances in medical intensive care unit patients: a randomized, controlled study. Crit Care. 2012;16:R56.

22. Iapichino G, Albicini M, Umbrello M, Sacconi F, Fermo I, Pavlovich R, et al. Tight glycemic control does not affect asymmetric-dimethylarginine in septic patients. Intensive Care Med. 2008;34:1843-50.

23. Kalfon P, Giraudeau B, Ichai C, Guerrini A, Brechot N, Cinotti R, et al. Tight computerized versus conventional glucose control in the ICU: a randomized controlled trial. Intensive Care Med. 2014;40:171-81.

24. McMullin J, Brozek J, McDonald E, Clarke F, Jaeschke R, Heels-Ansdell D, et al. Lowering of glucose in critical care: a randomized pilot trial. J Crit Care. 2007;22:112-8; discussion 8-9.

25. Mitchell I, Knight E, Gissane J, Tamhane R, Kolli R, Leditschke IA, et al. A phase II randomised controlled trial of intensive insulin therapy in general intensive care patients. Crit Care Resusc. 2006;8:289-93.

26. Oksanen T, Skrifvars MB, Varpula T, Kuitunen A, Pettila V, Nurmi J, et al. Strict versus moderate glucose control after resuscitation from ventricular fibrillation. Intensive Care Med. 2007;33:2093-100.

27. Preiser JC, Devos P, Ruiz-Santana S, Melot C, Annane D, Groeneveld J, et al. A prospective randomised multi-centre controlled trial on tight glucose control by intensive insulin therapy in adult intensive care units: the Glucontrol study. Intensive Care Med. 2009;35:1738-48.

28. Savioli M, Cugno M, Polli F, Taccone P, Bellani G, Spanu P, et al. Tight glycemic control may favor fibrinolysis in patients with sepsis. Crit Care Med. 2009;37:424-31.

29. Umpierrez G, Cardona S, Pasquel F, Jacobs S, Peng L, Unigwe M, et al. Randomized Controlled Trial of Intensive Versus Conservative Glucose Control in Patients Undergoing Coronary Artery Bypass Graft Surgery: GLUCO-CABG Trial. Diabetes Care. 2015;38:1665-72.

30. van den Berghe G, Wouters P, Weekers F, Verwaest C, Bruyninckx F, Schetz M, et al. Intensive insulin therapy in critically ill patients. N Engl J Med. 2001;345:1359-67.

31. Van den Berghe G, Wilmer A, Hermans G, Meersseman W, Wouters PJ, Milants I, et al. Intensive insulin therapy in the medical ICU. N Engl J Med. 2006;354:449-61.

32. Walters MR, Weir CJ, Lees KR. A randomised, controlled pilot study to investigate the potential benefit of intervention with insulin in hyperglycaemic acute ischaemic stroke patients. Cerebrovasc Dis. 2006;22:116-22.

33. Wang Y, Li JP, Song YL, Zhao QH. Intensive insulin therapy for preventing postoperative infection in patients with traumatic brain injury: A randomized controlled trial. Medicine (Baltimore). 2017;96:e6458.

34. Mohod V, Ganeriwal V, Bhange J. Comparison of intensive insulin therapy and conventional glucose management in patients undergoing coronary artery bypass grafting. J Anaesthesiol Clin Pharmacol. 2019;35:493-7.

35. Poole AP, Finnis ME, Anstey J, Bellomo R, Bihari S, Biradar V, et al. The Effect of a Liberal Approach to Glucose Control in Critically Ill Patients with Type 2 Diabetes: A Multicenter, Parallel-Group, Open-Label Randomized Clinical Trial. Am J Respir Crit Care Med. 2022;206:874-82.

36. Santana-Santos E, Kanke PH, Vieira RdCA, Oliveira LBd, Ferretti-Rebustini REdL, Menezes AFd, et al. Impacto do controle glicêmico intensivo na lesão renal aguda: ensaio clínico randomizado. Acta Paulista de Enfermagem. 2019;32:592-9.

37. Gunst J, Debaveye Y, Guiza F, Dubois J, De Bruyn A, Dauwe D, et al. Tight Blood-Glucose Control without Early Parenteral Nutrition in the ICU. N Engl J Med. 2023;389:1180-90.
