# Supplementary material for: The optimal glycemic target in critically ill patients: an updated network meta-analysis
Source: J Intensive Care. 2024 Apr 14;12:14. doi: 10.1186/s40560-024-00728-0 (PMC11017653; doi:10.1186/s40560-024-00728-0)
Supplement: Supplementary file 5 — Additional file 5. The network of all eligible comparisons for the meta-analysis. [file 40560_2024_728_MOESM5_ESM.docx]

**Additional file 5.** The network of all eligible comparisons for the meta-analysis.


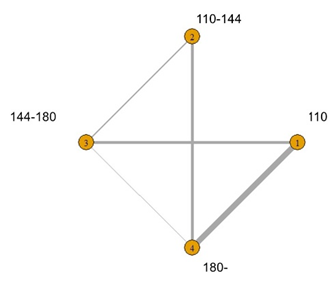
a Hospital mortality b 28 or 30 day mortality


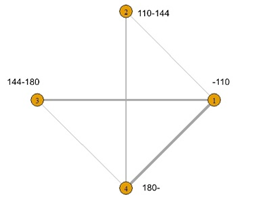


c Long-term mortality d Incidence of infection


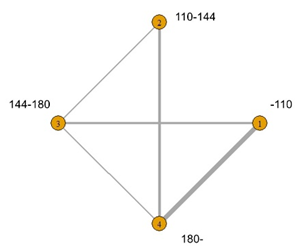

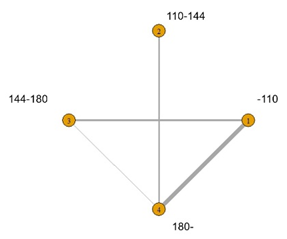


e Incidence of hypoglycemia. f Incidence of acute kidney injury


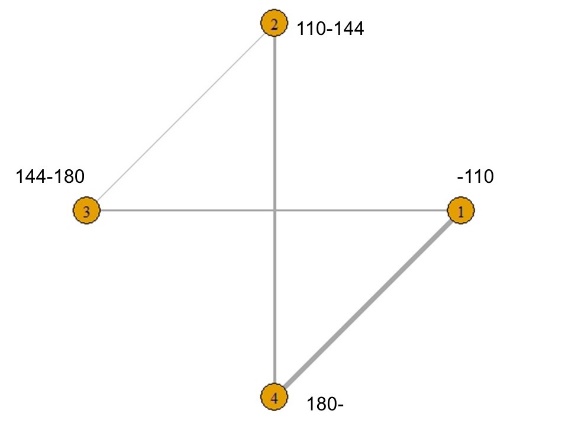

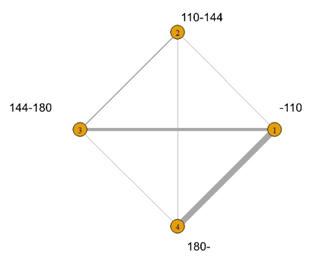


Lines represent direct comparisons.
