# Supplementary material for: The optimal glycemic target in critically ill patients: an updated network meta-analysis
Source: J Intensive Care. 2024 Apr 14;12:14. doi: 10.1186/s40560-024-00728-0 (PMC11017653; doi:10.1186/s40560-024-00728-0)
Supplement: Supplementary file 6 — Additional file 6. Estimates of effects, and certainty of the evidence according to blood glucose levels. [file 40560_2024_728_MOESM6_ESM.docx]

**Additional file 6.** Estimates of effects, and certainty of the evidence according to blood glucose levels.

|  | Hospital mortality | | | 28 or 30 day mortality | | | Long-term mortality | | | Infection | | | Hypoglycemia | | |
| --- | --- | --- | --- | --- | --- | --- | --- | --- | --- | --- | --- | --- | --- | --- | --- |
|  | Relative effect  (95% CrI)** | Certainty of the evidence | | Relative effect  (95% CrI)** | Certainty of the evidence | | Relative effect  (95% CrI)** | Certainty of the evidence | | Relative effect  (95% CrI)** | Certainty of the evidence | | Relative effect  (95% CrI)** | Certainty of the evidence | |
| **vs <110mg/dL** | | | | | | | | | | | | | | | |
| 110-144 mg/dL | 0.90  (0.54 to 1.52) | ⨁◯◯◯ Very low | | 1.19  (0.65 to 2.27) | ⨁◯◯◯ Very low | | 0.92  (0.39 to 2.15) | ⨁◯◯◯ Very low | | 0.77  (0.49 to 1.19) | ⨁◯◯◯ Very low | | 0.86  (0.15 to 5.08) | ⨁◯◯◯ Very low | |
| 144-180 mg/dL | 0.92  (0.63 to 1.21) | ⨁◯◯◯ Very low | | 0.94  (0.78 to 1.11) | ⨁◯◯◯ Very low | | 0.93  (0.78 to 1.09) | ⨁⨁◯◯ Low | | 0.89  (0.65 to 1.17) | ⨁⨁◯◯ Low | | 0.19  (0.08 to 0.42) | ⨁⨁◯◯ Low | |
| >180 mg/dL | 1.08  (0.90 to 1.34) | ⨁◯◯◯ Very low | | 1.02  (0.85 to 1.25) | ⨁◯◯◯ Very low | | 0.99  (0.87 to 1.15) | ⨁⨁⨁◯ Moderate | | 1.26  (1.03 to 1.6) | ⨁⨁⨁◯ Moderate | | 0.19  (0.10 to 0.33) | ⨁⨁◯◯ Low | |
| **vs 110-144mg/dL** | | | | | | | | | | | | | | |  |
| 144-180 mg/dL | 1.02  (0.56 to 1.74) | ⨁◯◯◯ Very low | 0.79  (0.41 to 1.48) | | ⨁◯◯◯ Very low | 1.01  (0.43 to 2.46) | | ⨁◯◯◯ Very low | 1.15  (0.73 to 1.81) | | ⨁◯◯◯ Very low | 0.22  (0.04 to 1.12) | | ⨁⨁◯◯ Low |  |
| >180 mg/dL | 1.21  (0.74 to 1.99) | ⨁◯◯◯ Very low | 0.86  (0.46 to 1.59) | | ⨁◯◯◯ Very low | 1.08  (0.46 to 2.57) | | ⨁◯◯◯ Very low | 1.65  (1.10 to 2.58) | | ⨁⨁⨁◯ Moderate | 0.22  (0.04 to 1.30) | | ⨁⨁◯◯ Low |  |
| **vs 144-180mg/dL** | | | | | | | | | | | | | | |  |
| >180 mg/dL | 1.18  (0.86 to 1.81) | ⨁◯◯◯ Very low | 1.09  (0.86 to 1.43) | | ⨁◯◯◯ Very low | 1.07  (0.88 to 1.33) | | ⨁⨁⨁◯ Moderate | 1.42  (1.06 to 2.05) | | ⨁⨁⨁◯ Moderate | 1.00  (0.38 to 2.65) | | ⨁⨁⨁◯ Low |  |
| CrI, credible interval; SUCRA, surface under the cumulative ranking | | | | | | | | | | | | | | |  |

**Additional file 6.** Estimates of effects, and certainty of the evidence according to blood glucose levels (Continue).

|  | Acute kidney injury | | |
| --- | --- | --- | --- |
|  | Relative effect  (95% CrI)** | Certainty of the evidence | |
| **vs <110mg/dL** | | | |
| 110-144 mg/dL | 0.57  (0.22 to 1.36) | ⨁◯◯◯ Very low | |
| 144-180 mg/dL | 1.11  (0.36 to 3.42) | ⨁◯◯◯ Very low | |
| >180 mg/dL | 1.11  (0.70 to 1.72) | ⨁◯◯◯ Very low | |
| **vs 110-144mg/dL** | | |  |
| 144-180 mg/dL | 1.91  (0.51 to 8.44) | ⨁◯◯◯ Very low |  |
| >180 mg/dL | 1.93  (0.89 to 4.54) | ⨁◯◯◯ Very low |  |
| **vs 144-180mg/dL** | | |  |
| >180 mg/dL | 1.00  (0.31 to 3.21) | ⨁◯◯◯ Very low |  |
