# Supplementary material for: The optimal glycemic target in critically ill patients: an updated network meta-analysis
Source: J Intensive Care. 2024 Apr 14;12:14. doi: 10.1186/s40560-024-00728-0 (PMC11017653; doi:10.1186/s40560-024-00728-0)

**Additional file 7.** Forest plot of pairwise comparison between intensive insulin therapy vs conventional care (more than 144mg/dL) for hospital or 90-days mortality in diabetes patients.


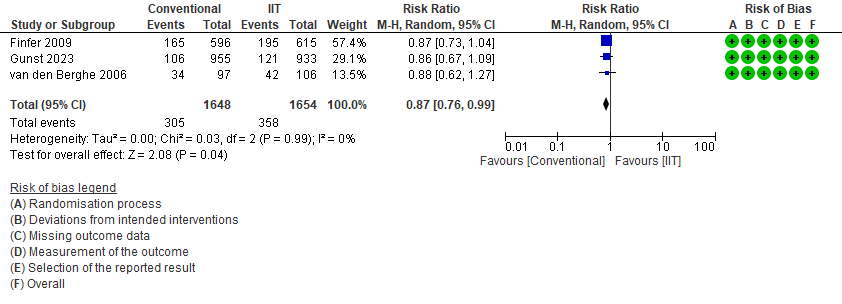

Supplement: Supplementary file 7 — Additional file 7. Forest plot of pairwise comparison between intensive insulin therapy vs conventional care (more than 144mg/dL) for hospital or 90-days mortality in diabetes patients. [file 40560_2024_728_MOESM7_ESM.docx]
